# Lower Limb Salvage Using the Medial Hemisoleus Flap Associated with the Reverse Sural Flap

**DOI:** 10.25122/jml-2019-0116

**Published:** 2019

**Authors:** Catalin Gheorghe Bejinariu, Silviu Adrian Marinescu

**Affiliations:** Department of Plastic and Reconstructive Surgery, “Bagdasar-Arseni” Emergency Clinical Hospital, Bucharest, Romania

**Keywords:** calf reconstruction, medial hemisoleus flap, reverse sural flap, calf infection

## Abstract

The paper aims to present the reconstructive surgical approach in the case of a patient with complex soft tissue lesions of the calf. The patient was the victim of a road accident resulting in the fracture of the right tibia for which screw-plate osteosynthesis was performed. The chosen therapeutic solution was represented by covering the soft tissue defects using a complex algorithm that involved the use of a reverse sural flap associated with a medial hemisoleus muscle flap and a split-thickness skin graft.

Considering functional recovery and the degree of patient satisfaction, the result of the therapeutic conduct was appreciated as very good. The association of the reverse sural flap with the medial hemisoleus flap can be a solution for solving complex cases with multiple soft tissue defects located in the middle and lower third of the calf.

## Introduction

Fractures of the calf caused by road accidents often raise problems regarding the reduction and fixation of the fragments through the use of osteosynthesis materials. The intensity of the traumatizing agent, the high degree of contamination, and the associated lesions represent negative prognostic factors, which must be carefully integrated into the therapeutic protocol to avoid the structural, septic, and functional complications.

The material presents the case of a 60 years old polytraumatized patient, the victim of a road accident that resulted in multiple injuries including minor cranial-cerebral trauma, thoracic and abdominal trauma with multiple rib fractures and fracture of the right tibia for which reduction and fixation were performed by using a plate and screws. The patient was admitted to the hospital 12 months after the trauma with calf infections associated with the exposure of the osteosynthesis material in the middle and inferior parts of the calf and local contamination with worms.

The therapeutic protocol consisted of surgical debridement in order to eliminate the worm contamination, in combination with the administration of the specific antibiotic therapy after obtaining the result from the antibiogram (gentamicin and a third-generation cephalosporin). The next step was the ablation of the osteosynthesis material, followed by the surgical reconstruction in one stage with a medial hemisoleus flap for the coverage of the soft-tissue defect located in the middle third of the calf and a reverse sural fasciocutaneous flap for the defect in the lower third of the calf, finally the muscular flap being covered by split-thickness skin graft.

The patient was reassessed periodically for 15 months, during the consults being determined that the flaps were integrated and that the functional recovery was completed, demonstrated by the socio-professional reintegration without limitations.

Eradication of the septic outbreak and worm contamination was achieved within seven days after the osteosynthesis material ablation by surgical excisional debridement, daily dressing changes with antiseptic solutions (chlorine-based compounds) and administration of a drug combination that included a third-generation cephalosporin and gentamicin.

**Figure 1:**
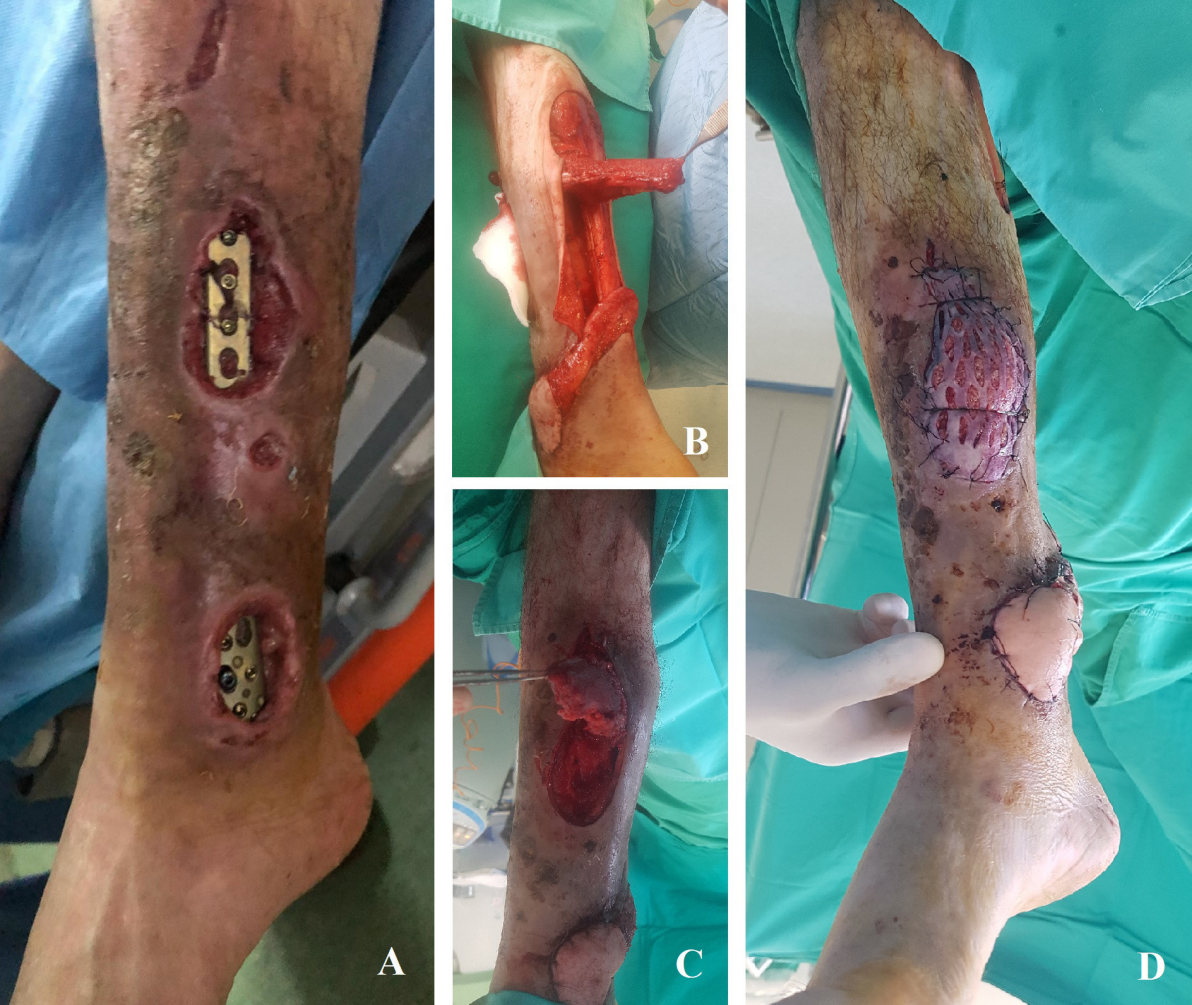
Hemisoleus flap used for the coverage of the defect located at the middle third of the calf **A.** Preoperative aspect; **B.** Dissected medial hemisoleus muscle flap; **C.** Tunneling the muscular flap; **D.** Immediate postoperative aspect.

A medial hemisoleus flap was used for the coverage of the soft-tissue defect from the middle third of the calf that was associated with bone exposure (Figure 1). In the next stage, the muscle was covered with a split-thickness skin graft harvested from the anterior face of the right thigh. Consequently, the postoperative evolution was favorable, with 95% graft integration, and the sutures were removed ten days after the surgical intervention.

**Figure 2:**
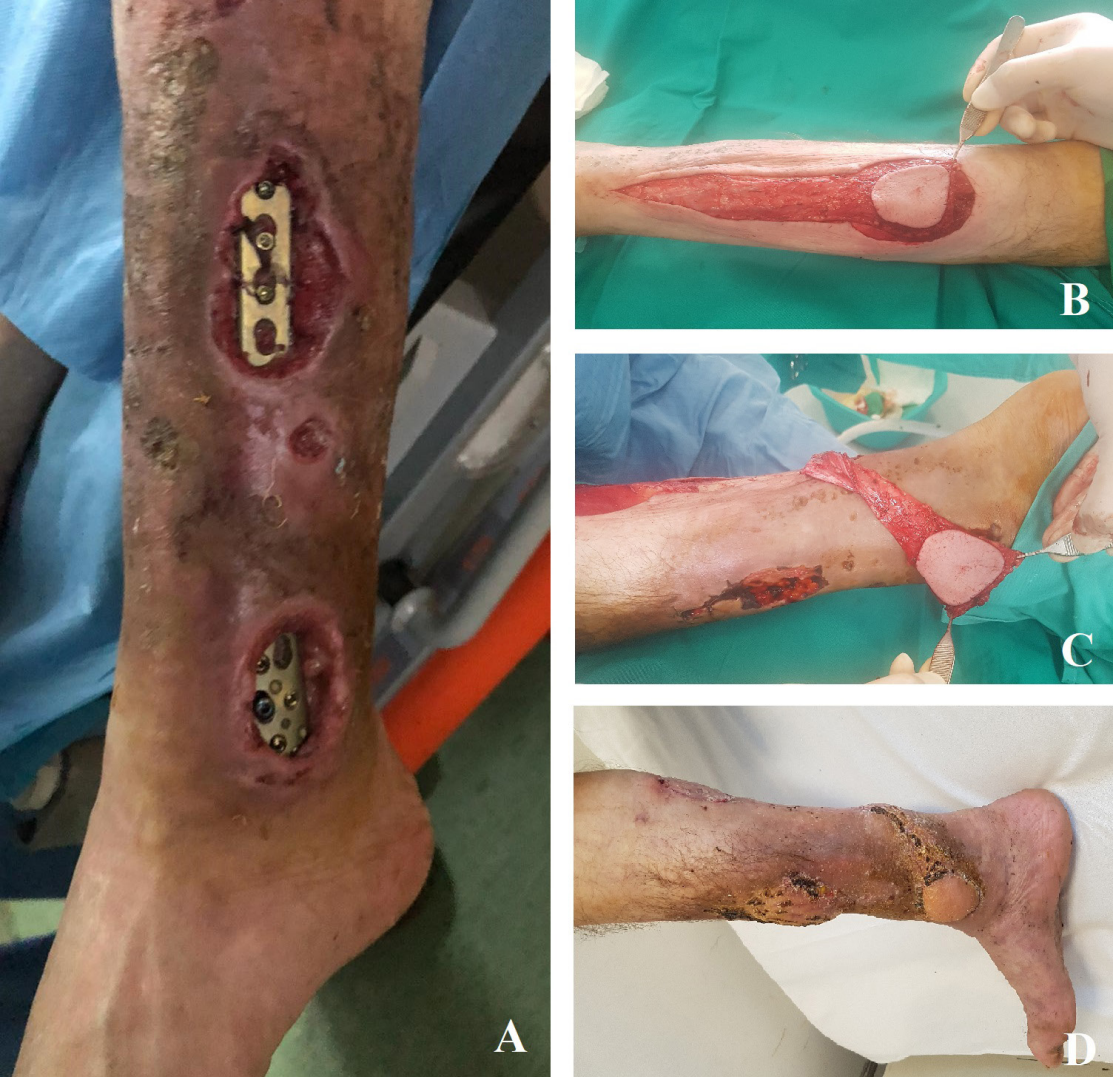
Sural flap used for the coverage of the defect located at the inferior third of the calf **A.** Preoperative aspect; **B.** Flap dissection; **C.** Soft tissue defect coverage; **D.** Postoperative aspect at 30 days

The soft tissue defect in the lower third of the calf was covered with a reverse sural fasciocutaneous flap, followed by grafting the donor site with a free split-thickness skin graft (Figure 2). The local evolution was favorable, the patient being discharged during the healing process.

**Figure 3:**
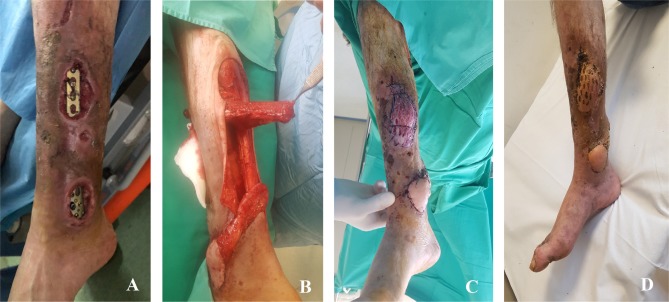
Reconstruction of the soft-tissue defects using the reverse sural and the medial hemisoleus flaps **A.** Preoperative aspect; **B.** Flaps dissection; **C.** Immediate postoperative aspect; **D.** Postoperative aspect at 30 days.

The hospitalization time in the plastic surgery department was 14 days, the patient being discharged during the healing process with a fully functional lower limb, subsequently being reassessed regularly for 15 months after the intervention. Postoperative follow-up showed that the flaps were fully integrated (Figure 3) without complications that would require surgical reintervention.

## Discussions

The reconstructive options for covering the soft-tissue defects of the calf have been carefully studied within the international scientific community, the specialized literature presenting a multitude of interesting articles in this respect. The current approach is to cover the defects with flaps based on the perforating arteries, which offers structural similarity with the anatomical region to be reconstructed, in the conditions of an increased safety profile and reduced morbidity of the donor site [1]. However, there are situations where reconstructive options are significantly limited by the intense contamination of the surrounding tissues, especially in rare cases when parasites are identified in the lesions. Despite the efforts, no feasible option was identified for performing the reconstruction through perforator flaps, therefore, given the existence of significant experience of the operating team regarding these techniques, the solution of using these versatile flaps proved to be the optimal choice [2].

The hemisoleus muscle flap represents a durable solution to cover the soft-tissue defects, especially in the lesions associated with bone exposure after the healing of a septic outbreak. The generous vascular component characteristic to this type of flap [3-6], constitutes without a doubt an advantage in terms of accelerating the healing process, creating the premises of a favorable outcome even in the conditions of subsequent osteitis [7]. The impairment of the functionality, characteristic of this kind of muscular flaps, did not prove to be an impediment in this case since the patient being able to move freely without difficulty at the evaluations performed for up to15 months.

The reverse sural flap represents a firm solution for the coverage of the soft-tissue defects located in the lower third of the pelvic limb [8], the reduced degree of difficulty of the harvest and the ability to associate with an extremely varied range of other reconstructive techniques contributing to the increased popularity of this type of flap [9-11]. However, it should be mentioned that in situations where it is possible, reconstruction using perforator flaps should be the first option [12-14]. The overall vision is an essential element that contributes to solving this kind of complicated cases [15]; in this sense the selection of the surgical technique is influenced by multiple factors, such as local features of the lesion and the patient, the reconstructive options available and, last but not least, the surgeon’s experience regarding the available techniques.

## Conclusions

The association of reconstructive techniques that involves the usage of the reverse sural flap and the hemisoleus flap is a feasible solution for solving cases that associate multiple soft tissue defects of the calf.

In this case, the use of the medial hemisoleus flap for the coverage of the soft-tissue defect located in the middle third of the calf was not associated with a significant impairment of the patient’s locomotor capacity, the patient being able to move normally and carry out his daily and professional activity without limitations.

The association between the hemisoleus muscle flap and the reverse sural flap may be a solution for complex cases where the use of perforator flaps is not possible.

Muscle flaps represent the “safety belt” in the cases that require complex reconstructions of the calf, often being the optimal solution for patients with complex fractures associated with neglected septic processes and soft tissue defects of considerable size.

## Conflict of Interest

The authors confirm that there are no conflicts of interest.
